# Remarks on Fistula in Ano

**Published:** 1847-10

**Authors:** William M. Boling

**Affiliations:** Montgomery, Alabama


ART. II.—*Remarks on Fistula in Ano.* By WILLIAM M. BOLING, M. D., of Montgomery, Alabama.

It has been for a long time a settled question among surgeons, that the operation by incision for fistula in ano is decidedly the most successful method of treatment—the only one, in short, in the present state of our knowledge which, except under peculiar circumstances, can be resorted to with propriety. Every other course of procedure is attended with great uncertainty, and even where a cure is the result, it is generally at the expense of protracted suffering, and much loss of time; while by the method of incision a cure may be promised, with almost as much certainty of success, and as promptly, as in any other affection requiring operative measures. By all it is admitted that a spontaneous recovery is a most rare occurrence. Notwithstanding the promptness and certainty of the cure in most instances following the operation, and simple as the matter at first glance may appear, the greatest diversity of opinion exists among the highest authorities, as to the manner in which the division of the parts exercises a curative influence. As a matter of curiosity merely, I have selected passages from the published opinions of several authors, in which this difference of sentiment is manifested, and which, taken together, render it evident, I think, that the explanation of neither is satisfactory. At the same time I have no explanation of my own to offer instead of the apparently untenable ones of the authors cited.

Perhaps the most popular and generally received explanation at present is, that, to the quiescence of parts induced by the division of the sphincter ani, is the cure mainly attributable.

Druitt says: "It is extremely difficult to heal, both because the constant contractions of the sphincter and levator ani interfere with the union of its sides, and because of the passage of fecal matter into it from the bowels." It will be recollected that the blind external fistula has no communication with the cavity of the intestine, and consequently that the latter of the two causes named in the above quotation can have no agency whatever in retarding the cure in such cases.

Again, the same author observes: "The grave remedy for this affection is division of the sphincter ani so as to prevent contraction of that muscle for a time, and cause the fistula to heal from the bottom."

Bush says: "The contraction of the sphincter, by separating the intestine from the walls of the pelvis will render reparation impossible. * * * To be short, as the object in dividing the rectum is to prevent contraction, and consequent separation from the walls of the pelvis, it is unnecessary to divide more than the portion which under ordinary circumstances is in a state of contraction; hence the incision ought not to extend higher than the upper edge of the internal sphincter."

Ferguesson, speaking of the division, observes: "Indeed with few exceptions the success of the operation seems chiefly to depend on this circumstance"—merely stating, in his opinion, a fact, but offering no explanation.

Samuel Cooper, in his 'First Lines,' observes: "The difficulty of healing many abscesses near the rectum, depends upon their being influenced by the action of the levator and sphincter ani muscles, which have a constant tendency to prevent the union of the granulations, and the coalescence of the sinus."

Sir Astley Cooper says: "From its vicinity to the rectum, it is influenced by the action of the sphincter and levator ani, and that these muscles have a constant tendency to prevent the union of the granulations and coalescence of the sinus. It therefore rarely happens but that the surgeon is required to assist nature by dividing the sphincter, and thus destroying its influence upon the sinus."

According to Pancoast, "The indications to be fulfilled are the laying open of the fistulous tracts and the sphincter muscle, which dams up the fecal matter, turns it into the cavity of the abscess, and keeps up such frequent motion of the parts as to prevent the tendency to heal." Here a more extended view is taken of the advantages to be derived from the operation.

Sir Charles Bell asks, "How high may the operator go with his incision of the rectum?" and answers, "It is needless to go higher than the sphincter, for it is the sphincter which prevents the fistula from healing."

Liston says: "The object of the surgeon is to widen the outlet for a time and incapacitate the sphincter muscle from acting at all."

Boyer, in explanation of the frequent degeneration of abscesses near the anus into fistulas, observes: "Ici en effet les partes de l'absces sont formés en dehors par le basin, qui ne peut rapprocher du rectum, qui tend sans cesse, à s'eloigner du basin, par l'effet de la contraction de ses fibres musculaires." He recommends the usual operation of course, but offers no explanation of the manner in which the obstacle to the cure which he alludes to is removed by it.

It will be seen that several of the authors above quoted, appear to attach as much importance to the motions of the levator ani in preventing a spontaneous cure, as to those of the sphincter, yet are perfectly satisfied with recommending the division of the latter, though at the same time this can have little or no effect in preventing the action of the former.

The result of two cases which I have treated would seem to show, that the mere motions of the sphincter ani muscle can have but little agency in retarding the cure, and that it is not principally upon the quiescence of parts resulting from the division of fibres that the success of the operation mainly depends. In the cases alluded to (both fistulæ being complete) the external opening was situated precisely in the mesial line, between the verge of the anus and the point of the coccyx. It did not occur to me till the moment of the introduction of the bistoury, in the first case, that owing to the situation of the fistula, and the necessary direction of the incision, the sphincter ani muscle could not be divided in the operation, but that its fibres would be merely separated, and, (the patient being a lady, who submitted to the operation with reluctance, and to whom I had given assurance of a speedy cure), I well recollect the anxiety the circumstance occasioned me, when I reflected that the quiescence of the parts, upon which I had counted for the cure, would not result from the operation. Notwithstanding the fact, however, that not a fibre of the sphincter muscle was divided in either case, the cure was complete in each before the termination of the third week from the day of the operation.

I will now make a few extracts from authors who offer other explanations of the mode of the curative influence of the operation.

Benjamin Bell, speaking of sinuses in general, says:—"Knowing that adhesion readily takes place between contiguous parts in a state of inflammation, we endeavor to make these parts inflame that we wish to adhere to each other." * * * "It may be accomplished either by the introduction of a cord of cotton or silk along the course of the sore, or by laying the sinus open through its whole length, so as to convert it as nearly as possible into the state of a recent wound."

Again; "The leading object to be kept in view in the treatment of sinuses, is the destruction or annihilation of the cavities from whence the matter is discharged." * * * * * "Where pressure can be employed, the sides of the sinuses are in some instances made to adhere by means of it; but in the fistula in ano this method of cure is indispensable." * * "The seton cannot be employed, for the irritation it would excite, would prove too severe a stimulus for the rectum, with which it would always be in contact."

"As in this situation, therefore, astringent and escharotic pastes and injections cannot be employed with safety, as pressure is inadmissible, and as cords of even the softest materials would excite a very insupportable degree of irritation; we are under the necessity of employing the only other remedy by which a degree of inflammation can be induced—a free and entensive incision along the whole course of the sinus." But surely it would seem, if the inflammation was all that was requisite, it might be induced, and of any desired degree, by regulating the strength of the astringent and escharotic pastes and injections used. There is some other element through which the curative agency of the operation is exercised so promptly, than the mere inflammation induced by it. It is a fact which must be admitted, however, that till modern times some of the principal agents in the treatment of fistula were astringent and caustic injections and ointments; and by the inflammation, or rather the altered action induced by their application, a cure was occasionally effected. Indeed, from a fact stated by Dionis (cours d'operations) it is probable that this took place eventually in a larger proportion of cases than at the present day we are taught to believe. "Il y á environ trente ans qu' a Paris un nommé Lemoyne *acquit une grosse reputation* pour la guérison des fistules. Sa methodé consistait dans l'usage du caustique." * * * "Ceux a qui le ciseau faisoit horreur se mettoient entre ses mains; et comme le nombre des poltrons est fort grand, il ne manquoit point de pratique."

In his Surgical Dictionary, Samuel Cooper says: "The surgery required in these cases, consists in laying open and dividing the sinus or sinuses in such a manner that there may be no possible lodgment for matter." It is however a fact, that many fistulas, and such too as will not heal without the operation, and are readily cured by it, from their direction, limited extent, and free external opening, afford no possible opportunity for the collection and retention of matter, which of necessity escapes as fast as it is secreted.

Larrey says: "It appears clear to me that when this orifice (the internal opening) is not included in the section of the bistoury, the fistula continues or returns. * * * * The whole success of the operation then depends on finding the internal orifice." But if we are to infer from this, that the author means that it is the internal opening mainly that prevents the healing of fistulæ, where no operation is performed, how are we to account for the indisposition to heal which exists in the blind external fistula? M. Roux, we are told, (Medico-Chirurgical Review, 1842,) "gives himself very little trouble about searching for the opening into the gut."

Sir Benjamin Brodie overcomes one principal difficulty as regards the explanation of the manner in which the incision so promptly effects a cure, by asserting that an internal opening always exists. "This," he says, "affords a very reasonable explanation of the formation of these abscesses; it is almost impossible to understand on any other ground why suppuration should take place in the vicinity of the rectum more than in any other part of the body, and why the cellular membrane there, should suppurate more than cellular membrane should elsewhere."

"This being the case it is easy to understand the reason why these abscesses do not heal. The least quantity of mucus even from the gut, or of feculent matter issuing into the abscess is sufficient to occasion irritation and prevent healing." * * * "Why is it that the abscess does not heal? It may, as I supposed formerly, partly arise from the unfavorable locality for healing in consequence of the muscular fibres of the parts being always in motion. The levator muscle and the sphincter are constantly drawing the parts asunder, so that they are not allowed to contract, but that is not a sufficient explanation. There is an internal opening to the abscess, and now and then a little bit of feces or mucus will become infiltrated into the cavity."

This would seem a very satisfactory explanation; one indeed to which no objection now suggests itself, were the position of the author, that an internal opening always exists, in accordance with the experience of other surgeons. In this, however, considering only the views of cotemporary writers, he stands almost alone, and admitting that he is incorrect in it, the explanation thereon based, falls of course at once to the ground. That Sir Benjamin Brodie is really in error in this respect, the positive testimony of many surgeons might be adduced. That of Boyer alone will suffice. Speaking of the blind external fistula he says:

"Plusieurs auteurs parmi lesquels ou en compte quelquesuns dont l'autorite est d'un grand poids en chirurgie, ont revoqué en doute l'existence de cette derniere espèce de fistules." * * * "J'ai partagé long-temps cette opinion, et je la partagerais peut etre encore si je n'avais en des autres preuves du contraire que le grand nombre de fistules que j'ai observé *sans pouvoir leur trouver d'orifice intense, et dans lesquelles l'incision du rectum* a eté suivie de la guérison; je pouvais penser que l'orifice fistuleux intense, rest sur un des bords de l'incision de l'intestin, n'a pas mis obstacle á la guérison et n'a donné lieu a la recidive de la fistule. Mais ayant eu occasion de dissequer le corps de plusieurs personnes mortes avec une fistule a l'anus, je me suis convaincu que chez quelques-unes ces fistules n'avaient aucune communication dans l'intestin."

Syme says: "When the fistula opens into the gut, more or less flatus and mucus must pass through it owing to the resistance which the sphincter muscles oppose to their exit by the anus, and thus adhesion or contraction in the surface of the sinus will be effectually prevented. But when the fistula is not complete, the reason why it should not heal like a sinus in any other part of the body is less apparent."

Mayo says: "It is customary to argue that the action of the sphincter externus ani is the cause which renders such an operation necessary." * * * "But the sphincter is habitually contracted, while the fistula on the other hand is a canal always open. The secretions and contents of the bowel, therefore, which are under the constant pressure of the adjacent parts have at once no escape through the anus, while they find a ready passage along the fistula, which the irritation they occasion prevents from closing.

"It is evident, however, that this account of the principle of the operation for fistula will not serve in that large class of cases in which there exists no communication between the fistula and bowel." * * * "Yet these cases are often as difficult to manage as the others are, and equally require the same operation for their relief.

"The true occasion why a fistula ani is so slow of closing is, that it is a sinus in the cellular tissue. In whatever part of the body such a sinus occurs, the same tediousness of healing is invariably observed."

The latter assertion in this last paragraph it seems is not corrobrated by the opinions of some others, for in the quotation just above from Syme, the remark is made that, "when the fistula is not complete the reason why it should not heal like a sinus in any other part of the body is less apparent." Nor does the explanation given in the first part of the paragraph, as to the reason why fistulæ are so slow of closing seem at all satisfactory, seeing that generally after the operation they are not difficult to heal, but on the contrary are promptly cured; yet the operation does not change the original character of the tissue in which they are seated—it is still cellular tissue.

The objections to the theory which attributes the little tendency of fistulæ to heal where no operation is performed, to the motions of the sphincter and levator ani muscles, and the benefits of the operation to the quiescence induced by it are:—first, that in the operation the levator ani at least is not rendered quiescent, and the motions of the anus are therefore but imperfectly controlled; second, in some cases—as in the two which I have named—and in all probability such cases are by no means rare—it is impossible that the motions of the sphincter even can be in the least degree impaired by the operation, seeing that not a single fibre of the muscle is divided in the section of the parts under such circumstances. And yet these cases heal as promply after the operation as others. It should be remembered too that in some instances the tract of the fistula does not pass from the internal to the external opening entirely outside of the sphincter muscle, but takes a course between its fibres, and though but a portion of the fibres of the muscle are divided in the operation, a cure, in these cases too is as certainly effected by it as in others.

This latter circumstance is equally applicable as an objection to the theory which attributes the difficulty,—as for instance in the opinion expressed by Bush, Boyer and others—not so much to the mere motions kept up by the sphincter, as to its constant contraction drawing the intestine, which forms a portion of the parietes of the fistula, from the side of the pelvis, which forms another portion; thus keeping the tract of the fistula always open, and its lining surfaces separate.

The objection to the view of Benjamin Bell, who seems to attribute the benefits of the operation to the degree of inflammation induced by it, is that by other means—as for instance stimulating injections—any degree of inflammation *may* be induced and kept up, by merely regulating the strength of the injection.

To the opinion of Samuel Cooper and others, which makes the difficulty to consist in the lodgment of matter, and the benefits of the operation to the provision of a free exit for it; it has already been objected, that there are some fistulæ requiring the operation, from which, from their free opening and direct course, the matter must of necessity be discharged as fast as it is secreted.

To the importance attached by Larrey, without any explanation however, to including the internal opening in the incision; and to the explanation of the manner in which the operation proves beneficial, by Brodie and others, who attribute the difficulty of cure to the passage of feces, flatus, and mucus into the fistula, in consequence of the contraction of the sphincter, it may be objected that the blind external fistulæ, in which this passage of feces into the cavity is impossible, equally demand, and are as promptly benefited by the operation, as those cases in which this is a possible and frequent occurrence.

Finally; it is no explanation of the manner in which the operation proves beneficial, to say that the reason why a fistula in ano will not heal without it, is that it is a sinus in the cellular tissue, unless this cellular tissue were changed by the operation into a different structure.

August 8th, 1847.

